# Supplementary material for: Predicting visual function by interpreting a neuronal wiring diagram
Source: Nature. 2024 Oct 2;634(8032):113–23. doi: 10.1038/s41586-024-07953-5 (PMC11446822; doi:10.1038/s41586-024-07953-5)
Supplement: Supplementary file 6 — CRF and ERF predictions for individual TmY4 and TmY9 cells. Analogous to Supplementary Data 3, but for TmY target types. Shown are the top four monosynaptic pathways, the strongest pathway passing through each of the top ten intermediary types (ranking from Extended Data Fig. 7), and the trisynaptic pathway Tm1–TmY–Dm3–TmY (see the section entitled Prediction of spatial normalization). [file 41586_2024_7953_MOESM6_ESM.zip › DataS4/TmY9q/720575940612350899.pdf]

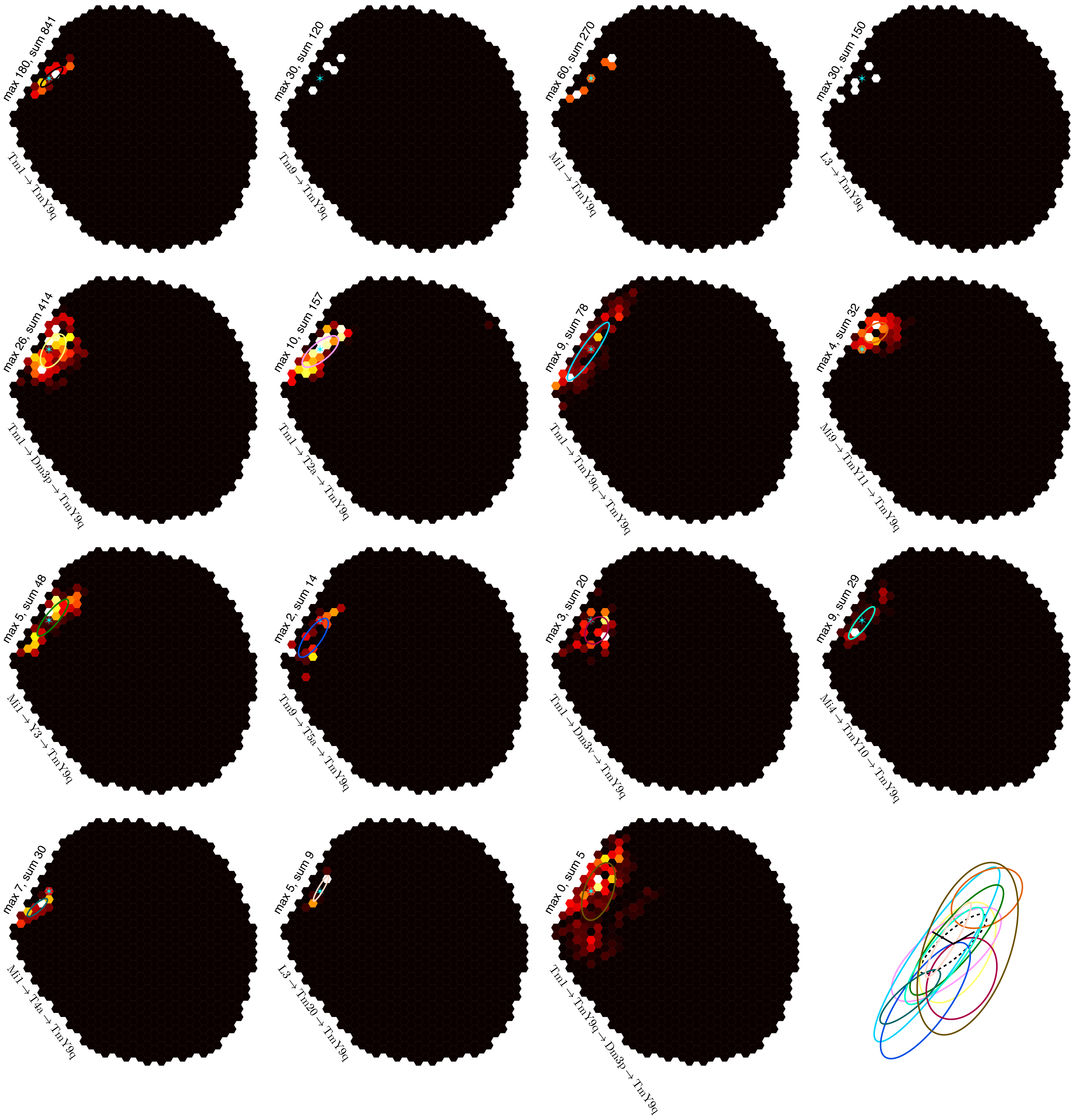

max 180, sum 841
Tm1 → TmY9q
max 30, sum 120
Tm9 → TmY9q
max 60, sum 270
Mi1 → TmY9q
max 30, sum 150
L3 → TmY9q
max 26, sum 414
Tm1 → Dm3p → TmY9q
max 10, sum 157
Tm1 → T2a → TmY9q
max 9, sum 78
Tm1 → TmY9q → TmY9q
max 4, sum 32
Mi9 → TmY11 → TmY9q
max 5, sum 48
Mi1 → Y3 → TmY9q
max 2, sum 14
Tm9 → T5a → TmY9q
max 3, sum 20
Tm1 → Dm3v → TmY9q
max 9, sum 29
Mi4 → TmY10 → TmY9q
max 7, sum 30
Mi1 → T4a → TmY9q
max 5, sum 9
L3 → Tm20 → TmY9q
max 0, sum 5
Tm1 → TmY9q → Dm3p → TmY9q
